# Determining Ricocheting Projectiles’ Temperature Using Numerical and Experimental Approaches

**DOI:** 10.3390/ma15030928

**Published:** 2022-01-25

**Authors:** Przemysław Badurowicz, Dawid Pacek

**Affiliations:** Department of Ballistics, Military Institute of Armament Technology, 05-220 Zielonka, Poland; pacekd@witu.mil.pl

**Keywords:** terminal ballistics, finite element method, thermal camera, identification of fire hazards

## Abstract

This paper describes the process of creating a numerical FEM (finite element method) model of the 5.56 × 45 mm SS109 projectile. The model was used to calculate the temperatures occurring in the projectile materials during the impact on the steel plate at an angle of 45°. The purpose of the investigation is to estimate the ability of a ricocheting projectile to cause ignition. For the same projectile, experimental tests were also carried out under the conditions adopted for the numerical investigation in order to validate the FEM model. During the experiment, temperature was measured with a thermal camera; the phenomenon was also recorded with a colour high-speed camera.

## 1. Introduction

Research on the flammability of various substances, such as forest litter, due to the use of firearms is justified by the large number of fires arising in military training areas. In Poland from 2008 to 2015, 475 fires with a total area of 2500 ha took place in the training areas. The average area of the fire was 5.26 ha, while in the remaining forests (state-owned and private) 0.25 ha; that is, in military areas they were several times larger [1]. There are also frequent media reports regarding wildfires caused by the use of firearms [2,3].

Following [4], the ignition of forest litter can be caused by temperatures of 200 °C ÷ 320 °C. According to [5], the ignition temperature of white birch, grass and shrub is 561 K (287.85 °C). If experimental tests and FE analyses confirm that the fragments reach the mentioned temperatures, it can be assumed that the risk of ignition is high.

Numerous papers on ignition and fire have been published [6,7,8,9,10,11,12,13,14,15], but the available sources show a shortage of references on fire ignition from ricocheting projectiles [16].

Research on the ability of various flammable substrates to ignite from ricocheting projectiles was carried out by the United States Department of Agriculture/Forest Service [16]. For the tests, intermediate ammunition: 7.62 × 39 mm, 5.56 × 45 mm and rifle ammunition: 7.62 × 51 mm, 7.62 × 54R was used. The following projectiles were used: with steel core, lead core, solid copper, with steel and copper jacket. The projectiles hit the inclined steel plate so that the ricochets fell into the box below. The box contained solid, flammable materials of organic origin, dried moss and peat. The most important conclusion from the research was that projectiles containing steel elements (core and jacket) and solid copper projectiles have the greatest ability to initiate a fire, and their fragments can reach 800 °C, which was recorded with a thermal camera. The debris formed after the ricochet of these projectiles was the largest so the heat loss was gradual, which facilitated ignition.

In [17], the authors measured the temperature of projectiles in flight with thermal cameras. The research concerned the 9 × 19 mm Parabellum pistol cartridge, the 0.38 Special revolver cartridge, the 7.62 × 39 mm intermediate cartridge and the 7.62 × 51 mm rifle cartridge. Temperatures observed for the projectiles ranged from 25 °C to 90 °C.

Numerous studies where the authors used FEM to determine the protective abilities of armour were found [18,19,20]. However, examples of the use of this method to determine the temperature of ricocheting projectiles were not encountered. On the other hand, there are studies [21] where the temperature of the impact of hard spherical steel particles into soft steel plates was determined using FE analyses. For impact velocity comparable to a rifle projectile (700 ÷ 1000 m/s), the temperature reaches up to 500 °C.

On the basis of the performed literature review it was found that there is a noticeable shortage of studies on the temperature measurement of projectiles hitting targets and their ricochets. It is also visible, in the case of the above mentioned phenomenon, that a research gap in using mutually verifying methods could authenticate the results. This is particularly important considering that the time between consecutive record images from thermal cameras (due to their hardware limitations) is many times higher than the time of contact of the projectile with the plate, when we can potentially expect the highest temperatures. The research presented in this article aims to fill this gap by showing a methodology in which observations by a thermal camera and a high-speed camera are also supplemented by the use of software for dynamic analysis.

**Figure 1:**
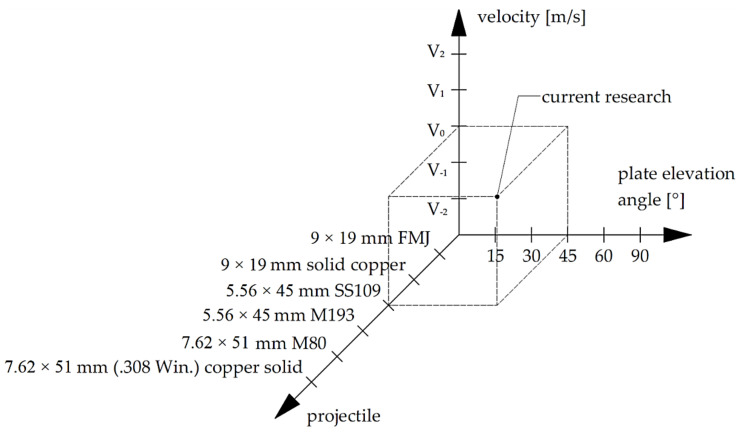
The scope of work to be performed in the project Arsonists (financing agreement number: DOB-BIO10/11/02/2019).

This article is an introduction to the work involving the examination of various types and calibres of ricocheting projectiles under different conditions (plate elevation angle and projectile velocity), for targets such as steel, concrete or granite plates (financing agreement number: DOB-BIO10/11/02/2019, acronym: Arsonists). The purpose is to check the ability of a ricochet to initiate fire on various types of substrates, including forest litter, peat, sawdust, or the initiation of combustion of flammable gases in the vicinity of a target, from which the projectile ricochets. This article focuses on the 5.56 × 45 mm SS109 projectile hitting a steel plate at an angle of 45 degrees (Figure 1).

Contrary to the work of [16], we decided to support the experimental research with FE analyses. Hence, it would be possible to recognise the phenomenon in more detail and calculate the ricochet temperatures, which will eventually help to determine if ignition of a given substance is possible. FEM is a useful tool, especially as thermal cameras have a large limitation in recording speed.

In case of a detailed analysis of the phenomenon of the possibility of ignition of a given material, the heat exchange between it and the fragmented parts of projectiles should also be taken into account, and it should be verified whether the time required for the tested medium to heat up to the ignition temperature is exceeded. Taking into account the above limitations, the recorded temperatures of projectile fragments should be treated when their values are below the ignition point of the material as temperatures that would certainly not cause ignition. If the temperatures of the fragments of the projectile exceed the limit values it should be understood, however, that the possibility of ignition exists, but it is not only determined by this fact.

As the underestimation of risk is more disadvantageous in the view of possible potential effects with regard to the assessment of fire hazards, the analysis of the results is simplified on the basis of the rigorous assumption that the temperature of the debris should not exceed the ignition point of the material concerned, and where this happens there is a risk of fire.

The objective of paper is to estimate if there is such risk and possibility of fire in the case of 5.56 × 45 mm HC (SS109) projectile impact, with nominal muzzle velocity, onto armour steel plates at an angle of 45 degrees. This paper also aims to present a research methodology where observations by a thermal camera are used together with a high-speed camera numerical simulation.

## 2. Development of a Numerical Model

**Figure 2:**
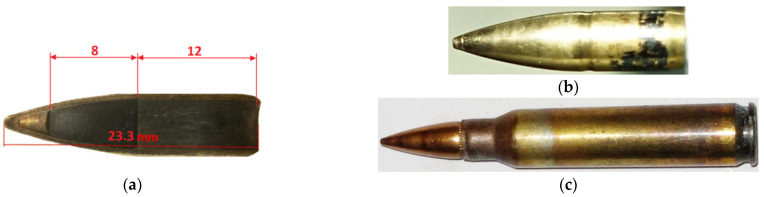
5.56 × 45 mm HC (SS109) ammunition used in the research: (**a**) cross-section [22], (**b**) whole bullet, (**c**) cartridge [18].

The numerical approach uses a 5.56 × 45 mm HC (SS109) Ruag full metal jacket (FMJ) projectile with a tombac jacket, a steel core in the front part of the bullet and a lead core in the rear part. The projectile and cartridge are shown in Figure 2, while the technical data is shown in Table 1. The same ammunition was used for the experimental tests.

**Figure 3:**
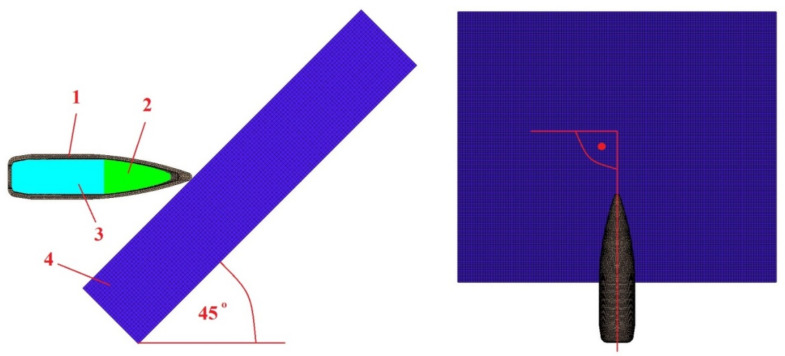
Numerical model of the 5.56 × 45 mm HC (SS109) projectile and Armox 600 plate: 1—tombac jacket, 2—steel core, 3—lead core, 4—Armox 600 armour plate.

The FEM mesh (division into finite elements) was made using HyperMesh 2017 software (Altair, Detroit, MI, USA). The numerical analysis was performed with Ansys Autodyn 2021 software (Ansys, Canonsburg, PA, USA). The developed FEM model is shown in Figure 3. The smooth particle hydrodynamics (SPH) meshless method was used for discretisation (0.1 mm distance between nodes) of the steel (number of nodes: 93,640) and lead (number of nodes: 40,271) cores, while for the jacket and the armour plate discretisation formulated in Lagrange’s description was used. A tetragonal (element size: 0.07 ÷ 0.66 mm) and hexagonal (element size: 0.4 mm) mesh was chosen for the jacket (number of elements: 43,956), and the armour plate (number of elements: 93,640), respectively. The plate adopted for calculations had dimensions of 50 × 50 × 10 mm (in the experiment 500 × 500 × 10 mm) to obtain a smaller number of mesh elements, which allowed for the shortening of the calculation time.

The following initial and boundary conditions were specified:

- The projectile impact velocity equal 987 m/s (value measured in the experiment);
- Fixing the Armox 600 armour plate on four side surfaces.

Moreover, material parameters and the connections between the projectile parts were defined.

The Johnson–Cook strength model relating to the flow of stresses, strains, strain rates and temperature distribution in the material was used. This model adopted for the jacket, steel core and armour plate, can be described by the following Equation (1) [18,24,25]:

(1) $$\sigma =\left[A+B\varepsilon^{n}\right]\times \left[1+C\ln\dot{\varepsilon^{*}}\right]\times \left[1-T^{*m}\right]$$

where: *σ*—yield stress, *A*—static yield stress, *B*—strain hardening coefficient, *ε*—equivalent plastic strain, *n*—strain hardening exponent, *C*—strain rate coefficient, $\dot{\varepsilon^{*}}=\varepsilon /\varepsilon_{0}$—dimensionless plastic strain rate, *ε*_0_—reference rate of plastic deformation (${\dot{\varepsilon }}_{0}=1s^{-1}$), $T^{*}=\frac{T-T_{p}}{T_{t}-T_{p}}$—homologous temperature (dimensionless value of temperature), *T*—temperature, *T_p_*—room temperature, *T_t_*—melting temperature, *m*—thermal softening exponent.

The Johnson–Cook model determines the deformation at failure with the following Equation (2) [16,24]. This model was adopted for the jacket, steel core and armour plate:

(2) $$\varepsilon^{f}=\left[D_{1}+D_{2}e^{D_{3}\sigma^{*}}\right]\times \left[1+D_{4}\ln\left(\dot{\varepsilon^{*}}\right)\right]\times \left[1+D_{5}T^{*}\right]$$

where: *ε_f_*—equivalent plastic strain at failure, *D*_1_, *D*_2_, *D*_3_, *D*_4_, *D*_5_—material constants, $\sigma^{*}=\frac{p}{\bar{\sigma }}$—pressure/stress measureless dependence, *p*—pressure, $\bar{\sigma }$—equivalent of the von Mises stress.

The failure occurs when parameter *D* achieves a value 1:

(3) $$D=\sum \frac{\Delta \varepsilon }{\varepsilon^{f}}$$

where: Δ*ε*—increment of the effective plastic strain.

For the lead core, the material model included in the Ansys Autodyn database was selected. The material data adopted for the numerical investigation are presented in Table 2.

## 3. Experimental Research

**Figure 4:**
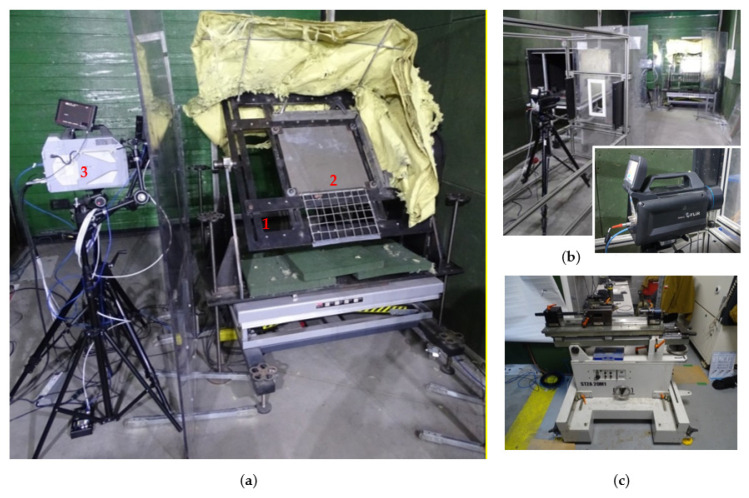
Laboratory stand for measuring temperature of ricocheting projectiles: (**a**) 1—mounting stand, 2—500 × 500 × 10 mm Armox 600 armour plate, 3—Photron Fastcam SA-Z 2100 K high-speed camera, (**b**) FLIR X6580sc thermal camera, (**c**) ballistic mount with 5.56 × 45 mm ballistic barrel.

**Figure 5:**
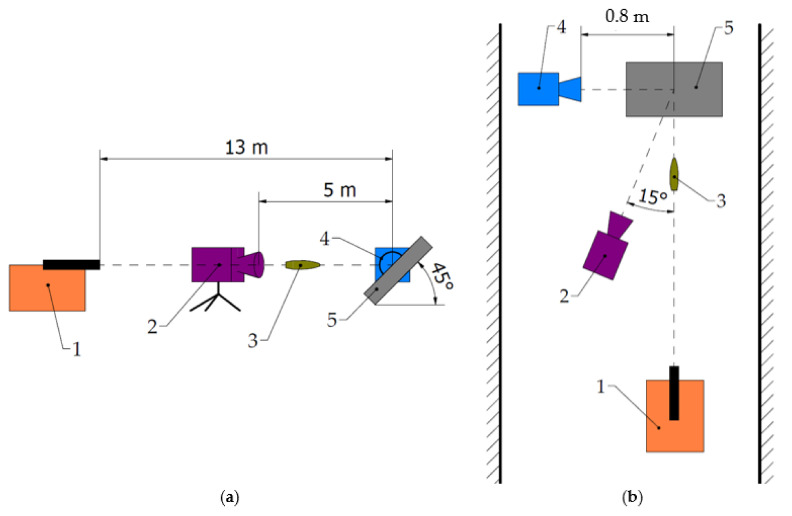
Schematic of the setup: (**a**) side view; (**b**) top view; 1—ballistic mount with barrel, 2—thermal camera, 3—projectile, 4—high-speed camera, 5—armour plate.

A mounting stand, for 500 × 500 × 10 mm Armox 600 armour plate, a Photron Fastcam SA-Z 2100 K high-speed camera, an FLIR X6580sc thermal camera and a 5.56 × 45 mm ballistic barrel were used for the experiment (Figure 4 and Figure 5). The armour plate was set at an elevation angle of 45°, causing an upward ricochet. In order to obtain the best quality image, it is beneficial to place the cameras (fast and thermal) as close as possible to the place of impact of the projectile. However, due to fragmented elements from the projectile and plate, there is a risk of damage to this apparatus. In the case of a high-speed camera, it is possible to protect it with transparent material such as polycarbonate (PC) or polymethyl methacrylate (PMMA) and record a given phenomenon without distortion. Therefore, the quick camera with protective cover was placed in the desired place during the research (beneficial in relation to the possibility of determining the moment of contact of the projectile with the armoured plate—about 80 cm from the place of impact perpendicular to the side surface/thickness of the steel plate). For a thermal camera, this was not possible because due to their insulating properties, the protective elements located in front of it would not allow it to record the actual temperatures. In order to not risk damaging the apparatus, and due to restrictions resulting from the specificity of the ballistic tunnel (width of approx. 3.3 m), the thermal camera was located at a safe distance at an angle of 15° in a horizontal plane to the point of impact and at the same height as the direction of the projectile’s flight path.

The high-speed camera and the thermal camera were used with recording speeds of 50,400 fps (frames per second) and 660 fps, respectively.

## 4. Results and Validation

**Figure 6:**
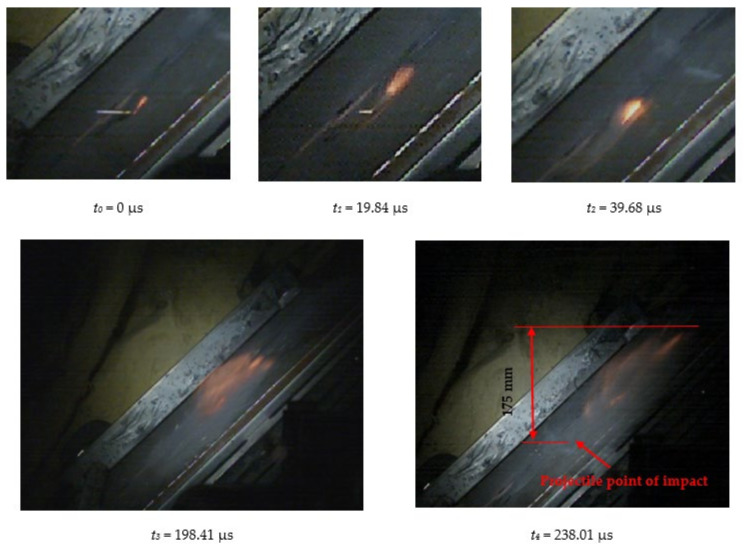
High-speed camera images of projectile impact onto armour plate.

Figure 6 shows the phenomenon of the projectile hitting the armour plate recorded with the high-speed camera. Three consecutive frames (*t*_0_–*t*_2_), one after another, are shown from the contact of the projectile with the plate to its complete fragmentation (“complete fragmentation” should be understood here as the situation when the most rear part of the bullet hits the plate or ricochets and it does not refer to the degree of fragmentation). The fourth frame shows the propagation of the debris.

The high-speed camera record (Figure 6) shows distorted proportions of the projectile; it is elongated. This is due to the relatively slow shutter speed; fast objects are then “blurred” but shortening this time would result in a recording that is too dark. In subsequent works, in order to eliminate this phenomenon it is planned to use more lighting, which would allow for a shorter shutter speed. The time for the first frame (*t*_0_) is 0 µs, and it is related to the moment of impact of the projectile on the armour plate and the beginning of the phenomena.

**Figure 7:**
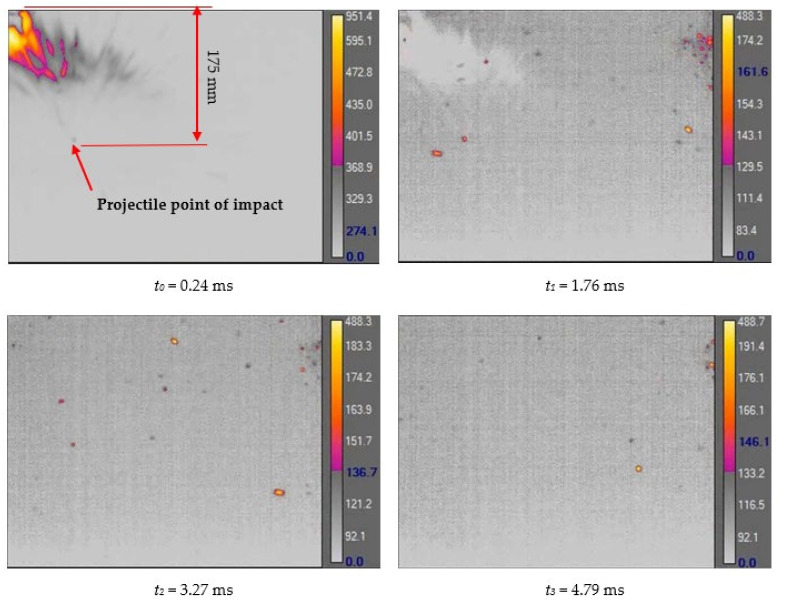
Thermal camera images of projectile impact onto armour plate.

Figure 7 shows the phenomenon using four successive frames, recorded by the thermal camera. The maximum temperature of 951.4 °C was recorded in the first frame (*t*_0_). Due to the low number of recorded frames per second it is impossible to determine based on a stand-alone thermal camera record (TCR) when in this recording the phenomenon started (the beginning of the contact of the projectile with the plate). The time of the first frame was set as 0.24 ms (238.01 µs), based on the height of the debris cloud. After measuring the value of this parameter in thermal imaging (175 mm) we investigated what time corresponded to it on the high-speed camera record (HSCR) (Figure 6 and Figure 7). The size of the debris cloud was measured by reading the number of pixels; the reference scale was based on the dimensions of the armour plate. The next two frames in TCR (*t*_1_, *t*_2_) show bullet fragments with a maximum temperature of 488.3 °C. The fourth frame (*t*_3_) shows the fragments with a maximum temperature of 488.7 °C. Due to the relatively low recording speed with the thermal camera (660 fps), the most important moment of the phenomenon was observed on only one frame of the recording (*t*_0_). 

Table 3 shows the projectile hitting the plate in numerical simulation. Projectile behaviour, fragmentation and a map of the temperature distribution for the projectile, its fragments and the armour plate for times as close as possible to the time recorded with the high-speed camera were presented, so that the calculations could be compared with the experiment. The height of the debris cloud at 238 µs for numerical approach is 187 mm, while for the experiment amounts it was 175 mm, which gives a difference equal to 12 mm (7%).

**Figure 8:**
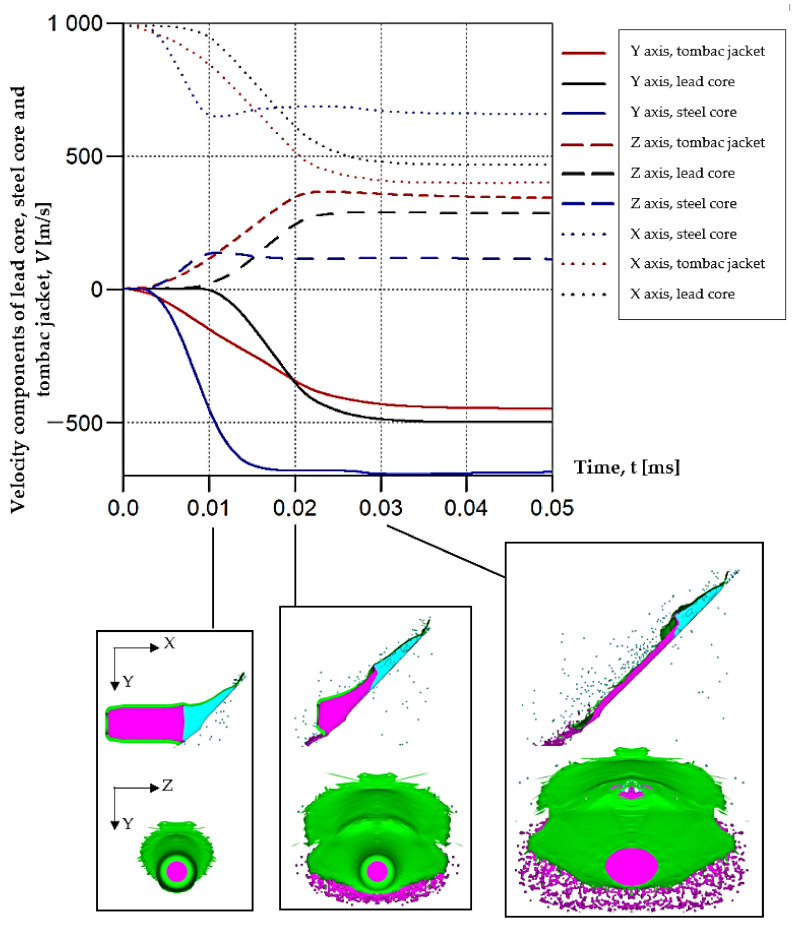
Velocity components of fragments of tombac jacket, steel core and lead core along the X, Y and Z axes.

Numerical simulations allow for an analysis of the components of average velocities of the fragments of projectiles along the OX, OY and OZ axes (Figure 8); in case of a high-speed or thermal camera, such analysis would be very difficult and time-consuming. This analysis allows for a better understanding of the phenomenon. The debris has the highest velocity along the OX and OY axes, with the highest values obtained for steel core fragments. The fragments along the OZ axis have the lowest velocity while at the same time the tombac jacket has the greatest velocity on this axis.

**Figure 9:**
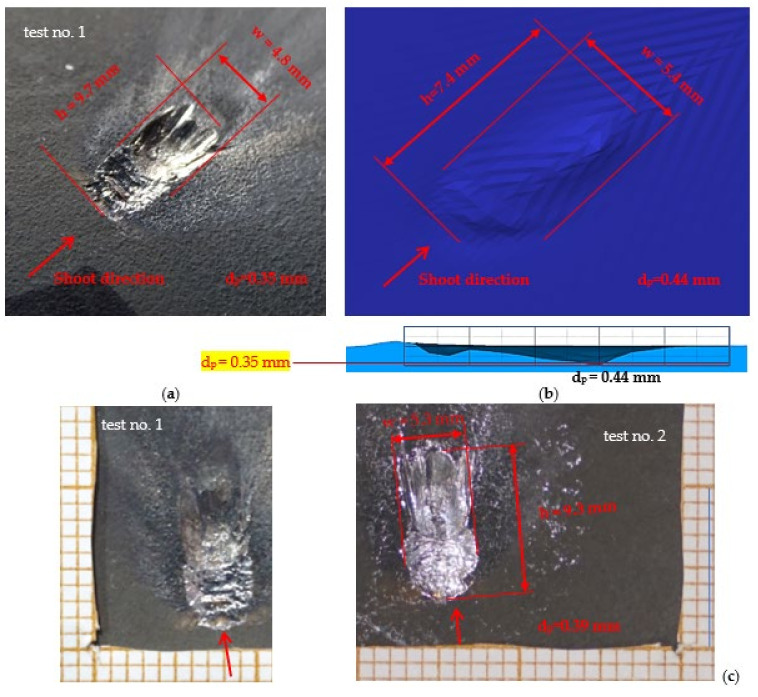
Trace after 5.56 × 45 mm HC (SS109) projectile impact onto Armox 600 armour plate: (**a**) experiment, (**b**) numerical simulation, (**c**) experiment—both tests (no. 1 and test no. 2).

The maximum temperature recorded during the calculations was above 3000 °C, so high temperatures were reached, however only by a few single finite elements. It is necessary to investigate these results in further works and check if this is a numerical issue (disturbance caused by extremely high deformations of finite elements) or if it represents a real phenomenon. It also seems advisable to look for methods that allow for recording the course of thermal phenomena with higher frequency of image recording. The Thermias system, which is a novelty on the market, allows the analysis of ultra-fast phenomena emitting visible thermal radiation. Thermias is based on any high-speed visible light camera, allowing temperature analysis to be carried out in accordance with the speed and resolution of a given camera. In future works, it is planned to use this system; efforts are currently being made in order to determine its availability. For the temperature distribution scale, the maximum value of 951.4 °C was assumed, i.e., the temperature obtained during the experiment. A relatively large number of projectile fragments with a temperature of 951.4 °C and higher were observed. Figure 9 shows the trace on the armour plate after the impact of the tested projectile during the experiment (Figure 9a) and obtained by the numerical approach (Figure 9b). Table 4 shows a comparison of the characteristic dimensions of the traces. Qualitative and quantitative similarity of both traces is noticeable.

To check the repeatability of the phenomenon the second experiment (test no. 2) was performed. Registering images from the thermal camera failed in this experiment. It was found, however, that deformations of armour steel plate obtained in both tests were very similar and the experiment performed gave quite repeatable results. In future works, more repetitions of the ballistic test for this variant will be performed.

For such a dynamic event characterised by very high object deformation and fragmentation, the authors have assessed the consistency between the results of the simulation and experiment to be sufficient. In the case of depth of penetration, 25% difference refers specifically only to the peak value. In Figure 9b it could be seen that in the numerical simulation a very small volume of indentation was below 0.35 mm depth obtained in the experiment. In the authors’ opinion, when assessing the compatibility of the simulation result with the experiment, the significance of the level of the measured value should also be taken into account (e.g., if the result of the experiment was 0.001 mm and the simulation result was equal to 0.002, it would give a difference from the study equal to 100%; however, it does not seem that the absolute difference in penetration depth equal to 0.001 would have any significance in relation to the phenomena studied). Therefore, in Table 4 there is an additional row in which the obtained difference in the maximum peak penetration depths is related to the plate thickness. Moreover, the shape of indentation in both cases (experiment and simulation) has a similar characteristic, where two areas could be distinguished: the one located from the direction of fire, which is flatter, and the other located in the further part with a steeper cross-section. In further work on the numerical model, however, an attempt will be made (e.g., reduction of finite element size) to obtain results with greater compatibility.

## 5. Conclusions

After carrying out the above investigations, the following conclusions can be drawn:

1. The developed numerical model reflects the actual behaviour of the projectile hitting the armour plate (bullet deformation and fragmentation), which was proven by comparing the frames from the recording with the high-speed camera to the appropriate FEM cycles (Figure 6, Table 3).
2. It is noticeable that the traces after a bullet impacts are very similar for the experiment and numerical investigation (Figure 9). The depths of penetration differ only by 0.09 mm, while the remaining characteristic dimensions differ by a maximum of 2.3 mm (Table 4), which is a satisfactory result for this type of dynamic phenomena.
3. For the high-speed camera recording, the height of the debris cloud at 238 µs is 175 mm, while in the simulation it is 187 mm. The difference is 12 mm (7%) (Figure 6, Table 3), which is a satisfactory result.
4. The maximum recording speed of the thermal camera is relatively low compared to the phenomenon under study. During long time intervals between the recorded frames, the maximum unregistered temperature of fragments may occur. It was recorded at the maximum speed of 660 fps and the time interval between consecutive frames was 1.5 × 10^−3^ s, while the contact of the projectile with the plate, during which the maximum temperature can be expected, lasts approximately 4 × 10^−5^ s.
5. The temperatures obtained during the experiment (951.4 °C) and numerical investigation (over 951.4 °C) prove the possibility of ignition of forest litter (ignition temperature 200 ÷ 320 °C) from ricocheting debris.
6. Numerical analysis, despite some deviations from the experiment, complements it and allows for a better understanding of the occurring phenomenon.
7. The developed numerical model will be used in a further part of the work to carry out a parametric analysis; it will be possible to check the influence of various variables on the temperature of a ricocheting projectile, such as bullet impact angle, impact velocity and plate material, e.g., concrete, granite, etc.
8. Performing parametric numerical analyses will allow us to limit the amount of experimental research and to obtain new information allowing for a better understanding of the studied phenomenon. As a result, it will facilitate the identification of hazards and the definition of extreme conditions for the initiation of a fire; hence, it will be possible to introduce appropriate procedures to prevent their occurrence.
9. By comparing projectile fragments and the temperature map (Table 3), it is evident that the highest temperature is achieved by steel fragments, which is consistent with the results presented in [16].
10. On the basis of numerical simulations, it can also be assumed that steel fragments move the greatest distance.

## Figures and Tables

**Table 1:** Technical data of ammunition used in the experiment [23].

Ammunition	Projectile Weight (*m_p_*)	Muzzle Velocity (*V*_0_)	Muzzle Energy (*E*_0_)
	g	m/s	J
5.56 × 45 mm HC (SS109) Ruag	4.0	945	1786
		^1^ 987	^2^ 1948

^1^ Value measured in the experiment, ^2^ value counted based on measured value.

**Table 2:** Material parameters adopted for numerical investigation [18,25,26].

Material	Johnson–Cook Strength Model					Johnson–Cook Failure Model				
	*A*, GPa	*B*, GPa	*C*	*n*	*m*	*D*_1_	*D*_2_	*D*_3_	*D*_4_	*D*_5_
Steel (core)	0.792	0.51	0.014	0.26	1.03	0.05	3.44	−2.12	0.002	0.61
Tombac	0.112	0.505	0.009	0.42	1.68	0.54	4.89	3.03	0.014	1.12
Armox 600 armour steel	1.58	0.958	0.00877	0.175	0.712	−0.4	1.5	−0.5	0	0

**Table 3:** Numerical simulation of projectile impact onto armour plate.

Time, µs	Materials Location		Temperature Distribution on Projectile and Armour Plate, Rear Side View of Projectile	Temperature Scale, °C
	Section of Plate and Projectile	Rear Side View of Projectile		
*t*_0_ = 0	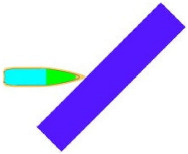	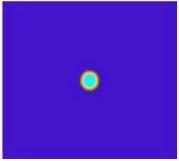	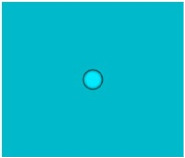	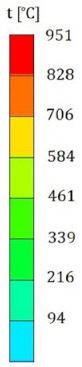
*t*_1_ = 20	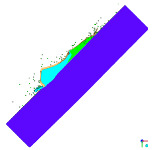	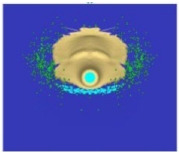	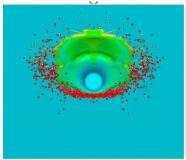	
*t*_2_ = 40	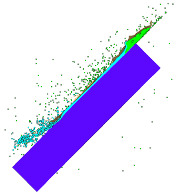	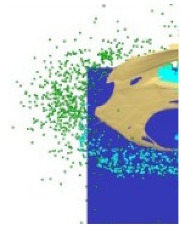	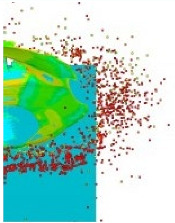	
*t*_3_ = 198	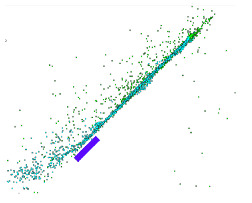	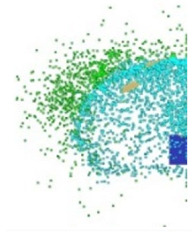	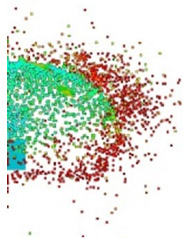	
*t*_4_ = 238	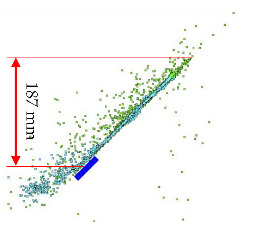	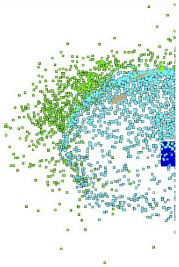	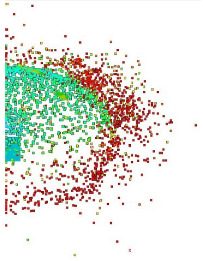	

**Table 4:** Dimensions of the trace after 56 × 45 mm HC (SS109) projectile impact onto Armox 600 armour plate.

	High (*h*)	Width (*w*)	Depth of Penetration (*d_p_*)
	mm	mm	mm
Numerical investigation	7.4	5.4	0.44
Experiment	9.7	4.8	0.35
Difference compared to the experiment	−2.3	+0.6	+0.09
Relative difference, %	−23.7	+12.5	+25.7
Difference between experiment and simulation referred to plate thickness	-	-	0.9%

## Data Availability

Not applicable.

## References

[1] Szczygieł R., Kwiatkowski M., Kołakowski B. (2017). Determining the Forest Fire Risk in Military Training Areas. Saf. Fire Tech..

[2] Are Environmentalists’ Anti-gun Policies to Blame for Wildfires in the West?. https://www.buckeyefirearms.org/are-environmentalists-anti-gun-policies-blame-wildfires-west.

[3] Guns Blamed for Sparking Some Wildfires in West. https://www.cbsnews.com/news/guns-blamed-for-sparking-some-wildfires-in-west/.

[4] Frandsen W.H. (1971). Fire spread through porous fuels from the conservation of energy. Combust. Flame.

[5] Koo E., Pagni P., Woycheese J., Stephens S., Weise D., Huff J. (2005). A Simple Physical Model for Forest Fire Spread Rate. Fire Saf. Sci..

[6] Stokes A.D. (1990). Fire Ignition by Copper Particles of Controlled Size. J. Electr. Electron. Eng..

[7] Rowntree G.W.G., Stokes A.D. (1994). Fire Ignition by Aluminum Particles of Controlled Size. J. Electr. Electron. Eng..

[8] Manzello S.L., Cleary T.G., Shields J.R., Yang J.C. (2006). On the ignition of fuel beds by firebrands. Fire Mater..

[9] Manzello S.L., Cleary T.G., Shields J.R., Yang J.C. (2006). Ignition of mulch and grasses by firebrands in wildland-urban interface fires. Int. J. Wildland Fire.

[10] Manzello S.L., Cleary T.G., Shields J.R., Maranghides A., Mell W., Yang J.C. (2008). Experimental Investigation of Firebrands: Generation and Ignition of Fuel Beds. Fire Saf. J..

[11] Linn R., Resiner J., Colman J.J., Winterkamp J. (2002). Studying wildfire behavior using FIRETEC. Int. J. Wildland Fire.

[12] Mell W., Jenkins M.A., Gould J., Cheney C. (2007). A physics-based approach to modeling grassland fires. Int. J. Wildland Fire.

[13] Yoshioka H., Hayashi Y., Masuda H., Noguchi T. (2004). Real-Scale Fire Wind Tunnel Experiment on Generation of Firebrands from a House on Fire. Fire Sci. Technol..

[14] Lee S.L., Hellman J.M. (1970). Firebrand Trajectory Study Using an Empirical Velocity-Dependent Burning Law. Combust. Flame.

[15] Woycheese J.P., Grayson S. (2001). Wooden disk combustion for spot fire spread. Proceedings of the Ninth International Conference INTERFLAM.

[16] Finney M.A., McAllister S.S., Maynard T.B., Grob I.J. (2016). A Study of Wildfire Ignition by Rifle Bullets. Fire Technol..

[17] Kerampran C., Gajewski T., Sielicki P.W. (2020). Temperature Measurement of a Bullet in Flight. Sensors.

[18] Wiśniewski A., Pacek D. (2015). Flexible Modular Armour for Protection Against the 5.56 × 45 mm SS109 Projectiles. Probl. Mechatron. Armament Aviat. Saf. Eng..

[19] Burian W., Żochowski P., Gmitrzuk M., Marciarz J., Starczewski L., Juszczyk B., Magier M. (2019). Protection effectiveness of perforated plates made of high strength steel. Int. J. Impact Eng..

[20] Fras T., Roth C.C., Mohr D. (2019). Dynamic perforation of ultra-hard high-strength armor steel: Impact experiments and modelling. Int. J. Impact Eng..

[21] Molinari J.F., Ortiz M. (2002). A study of solid-particle erosion of metallic targets. Int. J. Impact Eng..

[22] Adams B. (2003). Simulation on Ballistic Impacts on Armored Civil Vehicles. Master’s Thesis.

[23] Website of RUAG Company. https://www.ruag.com/system/files/2016-2/High_Performance_Infantry_Ammunition_en.pdf.

[24] Kacalak W., Królikowski T., Rypina Ł. (2013). Modeling of stress and displacement of the material in the difficult-Microcuting zone using the LS-DYNA software. Mechanic.

[25] Johnson G., Cook W. A constitutive model and data for metals subjected to large strains, high strain rates and high temperatures. Proceedings of the 7th International Symposium on Ballistics.

[26] Nilsson M. (2003). Constitutive Model for Armox 500T and Armox 600T at Low and Medium Strain Rates.

